# Leveraging a Fluorescent Fatty Acid Probe to Discover Cell-Permeable Inhibitors of Plasmodium falciparum Glycerolipid Biosynthesis

**DOI:** 10.1128/spectrum.02456-22

**Published:** 2022-10-31

**Authors:** Christie Dapper, Jiapeng Liu, Michael Klemba

**Affiliations:** a Department of Biochemistry, Virginia Tech, Blacksburg, Virginia, USA; Hebrew University of Jerusalem

**Keywords:** malaria, *Plasmodium falciparum*, fatty acid, neutral lipid, phospholipid, fluorescent probe, Malaria Box

## Abstract

A sensitive and quantitative fluorescence-based approach is presented for characterizing fatty acid acquisition and lipid biosynthesis by asexually replicating, intraerythrocytic Plasmodium falciparum. We show that a BODIPY-containing, green-fluorescent fatty acid analog is efficiently and rapidly incorporated into parasite neutral lipids and phospholipids. Prelabeling with a red-fluorescent ceramide analog permits normalization and enables reliable quantitation of glycerolipid labeling. Inhibition of lipid labeling by competition with natural fatty acids and by acyl-coenzyme A synthetase and diacylglycerol acyltransferase inhibitors demonstrates that the fluorescent fatty acid probe is acquired, activated, and transferred to lipids through physiologically-relevant pathways. To assess its utility in discovering small molecules that block parasite lipid biosynthesis, the lipid labeling assay was used to screen a panel of mammalian lipase inhibitors and a selection of compounds from the “Malaria Box” anti-malarial collection. Several compounds were identified that inhibited the incorporation of the fluorescent fatty acid probe into lipids in cultured parasites at low micromolar concentrations. Two contrasting profiles of suppression of neutral lipid and phospholipid synthesis were observed, which implies the inhibition of distinct pathways.

**IMPORTANCE** The human malaria parasite Plasmodium falciparum relies on fatty acid scavenging to supply this essential precursor of lipid synthesis during its asexual replication cycle in human erythrocytes. This dependence on host fatty acids represents a potential vulnerability that can be exploited to develop new anti-malarial therapies. The quantitative experimental approach described here provides a platform for simultaneously interrogating multiple facets of lipid metabolism- fatty acid uptake, fatty acyl-CoA synthesis, and neutral lipid and phospholipid biosynthesis- and of identifying cell-permeable inhibitors that are active *in situ*.

## INTRODUCTION

Malaria exacts an enormous toll on global public health. According to the World Health Organization’s World Malaria Report, an estimated 241 million cases and 627,000 deaths were attributed to malaria in 2020 (1). The human malaria parasite Plasmodium falciparum is responsible for most malaria-related mortality. All parasites rely to some extent on their hosts to provide key metabolic precursors and this creates vulnerabilities that can be exploited for the development of novel therapeutic agents and strategies. P. falciparum causes disease as it replicates within the host’s red blood cells, where it has access to a rich supply of metabolites in the plasma.

Fatty acids are essential metabolic building blocks for the synthesis of parasite lipids and are acquired from the host by intraerythrocytic asexual parasites (2–5). Interfering with fatty acid acquisition would be a promising avenue for anti-malarial development, yet we know relatively little about this process. The P. falciparum genome encodes 12 acyl-coenzyme A (CoA) synthetase homologs (6), a subset of which is exported into the host erythrocyte (7, 8), presumably to ensure that exogenous fatty acids are efficiently captured as acyl-CoA thioesters. The mechanism for subsequent transfer of fatty acyl groups across the parasite plasma membrane is currently unknown. It is also possible that fatty acids diffuse or are transported across the parasite plasma membrane and are activated to acyl-CoA within the parasite.

Vigorous rates of phospholipid synthesis are required to support growth of parasite plasma and organellar membranes, resulting in a 6-fold increase in phospholipid content in the mature, parasite-infected erythrocyte (9). Phosphatidylcholine (PC) and phosphatidylethanolamine (PE) are the major malarial phospholipids, present at 40 to 50% and 35 to 40% of total phospholipids, respectively (9). The CDP-choline (Kennedy) pathway for *de novo* synthesis of phospholipids is essential for parasite growth (10).

P. falciparum also produces significant quantities of the neutral lipids diacylglycerol (DAG) and triacylglycerol (TAG) (4, 5, 11, 12), which are deposited in lipid droplets (12, 13). These may serve as a form of temporary fatty acid storage and may also catalyze the crystallization of heme into hemozoin in the food vacuole (12–14). Synthesis of TAG appears to be essential, as the gene encoding diacylglycerol acyltransferase cannot be knocked out in P. falciparum (15).

While much work over the past several decades has provided a detailed picture of lipid biosynthetic pathways in P. falciparum, the extent to which the processes of fatty acid acquisition and *de novo* lipid biosynthesis are “druggable” remains largely unexplored. We sought to develop an assay that would permit a quantitative assessment of fatty acid uptake and incorporation into the dominant parasite neutral lipids (DAG and TAG) and phospholipids (PC and PE) of P. falciparum. Fatty acid analogs containing the green dipyrrometheneboron difluoride (BODIPY) fluorophore are attractive for this purpose as they are nonpolar, display high quantum yield and low environmental sensitivity, and are commercially available (16). BODIPY-fatty acid probes are efficiently incorporated into lipids in yeast and mammalian cells and have been used to develop screens for inhibitors of lipid biosynthesis in these organisms (17–21). As an internal control, we investigated the utility of a red-fluorescent ceramide analog, BODIPY-TR-ceramide, which labels the parasitophorous vacuole and red blood cell membranes (22, 23). We employed the quantitative, two-color assay to conduct a screen for inhibitors of P. falciparum lipid biosynthesis from two sources: a collection of fatty acid amide hydrolase and monoacylglycerol lipase inhibitors, and a subset of compounds from the “Malaria Box” anti-malarial collection.

## RESULTS

### Development of a quantitative, normalized assay for fluorescent fatty acid incorporation into parasite lipids.

We tested the ability of five fluorescent fatty acid analogs to serve as probes of fatty acid uptake and lipid biosynthesis in cultured P. falciparum (see Fig. S1 for structures). Synchronized trophozoite cultures (28 to 34 h post-invasion) were used for uptake experiments because synthesis of neutral lipids and phospholipids takes place during this stage as the parasites prepare for daughter merozoite formation. Each fatty acid was provided to parasite cultures at 30 μM in incomplete RPMI supplemented with 2 mg/mL fatty acid-free bovine serum albumin (BSA), with incubation for 1 h in a CO_2_ incubator at 37°C with gentle mixing. Parasites were isolated by saponin treatment, lipids were extracted with chloroform/methanol, and probe incorporation was assessed by two-stage thin-layer chromatography (TLC) to resolve neutral lipids and phospholipids. After each stage, fluorescence was imaged using a Typhoon flatbed scanner.

**FIG 1:**
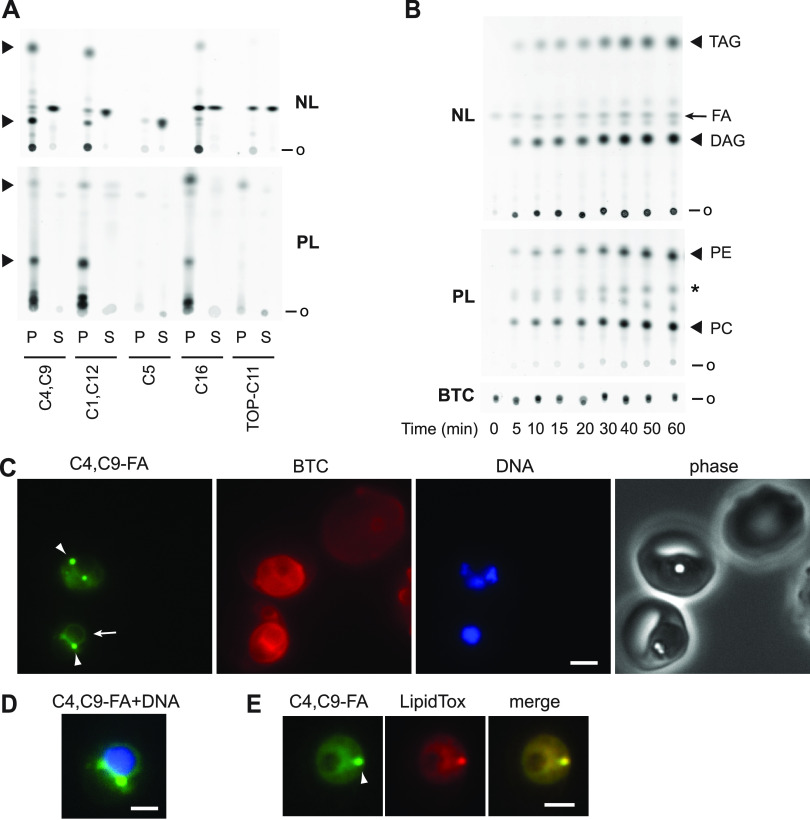
Labeling of parasite lipids with fluorescent fatty acid analogs. (A) Comparison of labeling of neutral lipids (NL) and polar lipids (PL) by five fluorescent fatty acids. P, parasite lipids; S, fluorescent fatty acid standard. Major lipid species are indicated with arrowheads. o, origin. (B) Time course of C4,C9-FA incorporation into parasite neutral and polar lipids. Presumed identities of major lipids species are indicated. FA, C4,C9-FA; *, residual DAG fluorescence; BTC, BODIPY-TR-ceramide fluorescence. (C) Live-cell images of two infected erythrocytes (center and lower left) and one uninfected erythrocyte (upper right) after labeling with BTC and C4,C9-FA. DNA was stained with Hoechst 33342. Arrow, perinuclear ER fluorescence; arrowheads, punctate fluorescence. (D) Merge of the C4,C9-FA and DNA panels from the lower left parasite in (C). The contrast of the C4,C9-FA panel was adjusted to emphasize the circumnuclear fluorescence, leading to saturation of pixels associated with the punctate fluorescence. (E) HCS LipidTox Red staining of neutral lipids in a parasite labeled with C4,C9-FA identifies the fluorescent punctum as a lipid droplet (arrowhead). Scale bar, 3 μm in C and E, 1.8 μm in D.

Robust incorporation into both neutral and polar lipids was observed with BODIPY-C1,12 and BODIPY-C4,C9 probes, while BODIPY-C16 was preferentially incorporated into polar lipids (Fig. 1A). In comparison with the BODIPY-labeled fatty acids, TOPFLUOR-C11 was marginally incorporated into parasite lipids, possibly due to steric differences with respect to the orientation of the fluorescent group relative to the fatty acyl chain (Fig. S1). BODIPY-C5, a medium-chain fatty acid analog, was not incorporated into any lipids. All subsequent experiments were conducted using the BODIPY-C4,C9 fatty acid analog (abbreviated C4,C9-FA) as this probe afforded efficient labeling of nonpolar and polar lipids.

Fatty acid diffusion across the erythrocyte membrane is rapid (24, 25), and while the erythrocyte cannot synthesize phospholipids *de novo*, it can incorporate exogenous fatty acids into triacylglycerol and phospholipids, the latter through the acyl editing pathway known as the Lands cycle (26–28). Thus, we asked whether any of the observed lipid labeling can be attributed to the host erythrocyte. A comparison of lipids extracted from equivalent numbers of uninfected and parasite-infected erythrocytes incubated with C4,C9-FA for 1 h revealed that uninfected erythrocyte lipids are not detectably labeled under these conditions (Fig. S2). Thus, the labeled lipids observed in Fig. 1A are presumed to originate from parasite metabolic pathways.

A time course of C4,C9-FA fatty acid labeling revealed its appearance in parasite neutral and polar lipids within minutes, with incorporation increasing in an approximately linear fashion over an hour (Fig. 1B, S3). Live-cell images of trophozoite-stage parasites (28 to 34 h post-invasion) after a 30 min labeling period revealed diffuse fluorescence across the parasite as well as perinuclear fluorescence suggestive of the endoplasmic reticulum (29), presumably due to incorporation into phospholipids generated through the Kennedy pathway (Fig. 1C, D). In addition, one to three intensely labeled punctate structures was observed that began to appear from ~30 h post-invasion (Fig. 1C). This punctate fluorescence colocalized with the neutral lipid stain HCS LipidTox Red (Fig. 1E; Pearson’s correlation coefficient = 0.93 ± 0.03, *n* = 8 puncta). This colocalization strongly suggests that these structures are lipid droplets, which develop as the asexual parasite matures (13). Notably, C4,C9-FA fluorescence was absent from images of uninfected erythrocytes (Fig. 1C), which is consistent with the lack of labeling observed by TLC analysis (Fig. S2).

Accurate quantitation of C4,C9-FA incorporation would benefit from an internal standard for normalization across samples. We found that the red-fluorescent ceramide analog BODIPY-TR-ceramide (BTC; Fig. S1) provided an orthogonal probe that could be extracted and quantified along with C4,C9-FA-labeled lipids. Consistent with previous reports (22, 23), a 1-h treatment of parasite cultures with 1 μM BTC was sufficient for robust labeling of parasite lipids in a diffuse distribution suggestive of incorporation into the parasitophorous vacuole and internal parasite membranes (Fig. 1C). Uninfected erythrocyte membranes were also labeled, albeit less intensely (Fig. 1C). After lipid extraction and TLC development of neutral lipids, BTC fluorescence remained at the origin and was quantified for normalization (Fig. S4A). Separation of polar lipids during the second TLC stage indicated that most of the recovered BTC was unmodified but did reveal the presence of a minor species (Fig. S4B), which is likely sphingomyelin due to its greater polarity (lower mobility) and prior evidence for its formation from a fluorescent ceramide analog (30). No cross talk was observed between the green and red fluorescent channels (data not shown).

**FIG 2:**
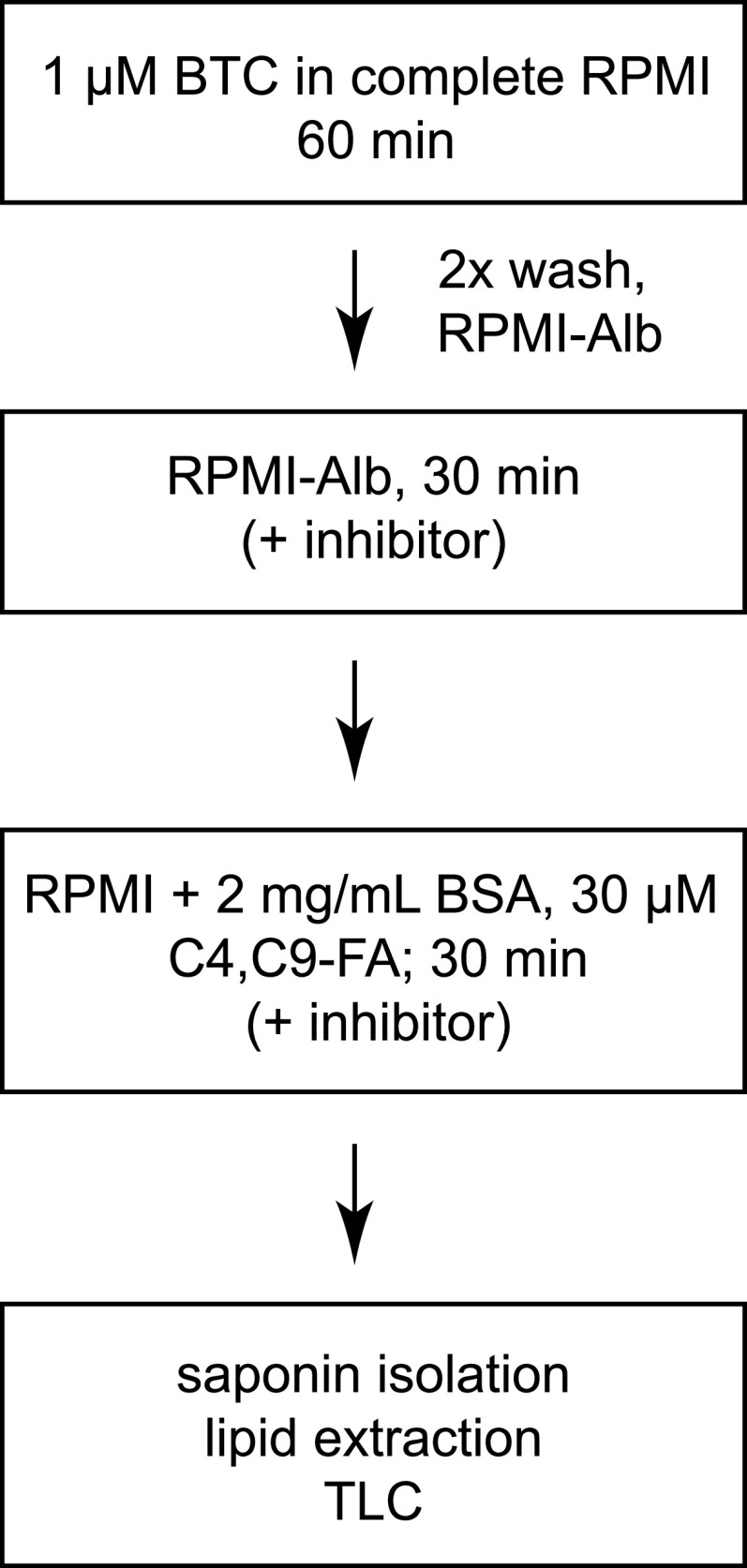
Standard protocol for C4,C9-FA labeling of parasite lipids in cultures of synchronized trophozoites. RPMI-Alb, incomplete RPMI; BSA, fatty acid-free bovine serum albumin.

The standardized workflow for normalized, quantitative analysis of C4,C9-FA incorporation into P. falciparum lipids is depicted in Fig. 2. First, the bulk culture is labeled with the internal standard BTC. Parasites are washed into incomplete RPMI and incubated for 30 min to deplete endogenous fatty acids and, when testing small molecules for inhibition, to permit their diffusion into cells and target engagement. C4,C9-FA/BSA is added to the parasite culture to initiate labeling. At the end of the 30 min incubation period, labeling is quenched by transferring cultures to nine volumes of cold PBS with 0.03% saponin. To test whether parasites remain viable throughout the assay, at the end of the C4,C9-FA labeling period the culture medium was replaced with compete RPMI and parasites were cultured to the next replication cycle. No loss of parasite viability was detected (data not shown).

### Assay validation: inhibition of diacylglycerol acyltransferase and acyl-CoA synthetase and competition by natural fatty acids.

**FIG 3:**
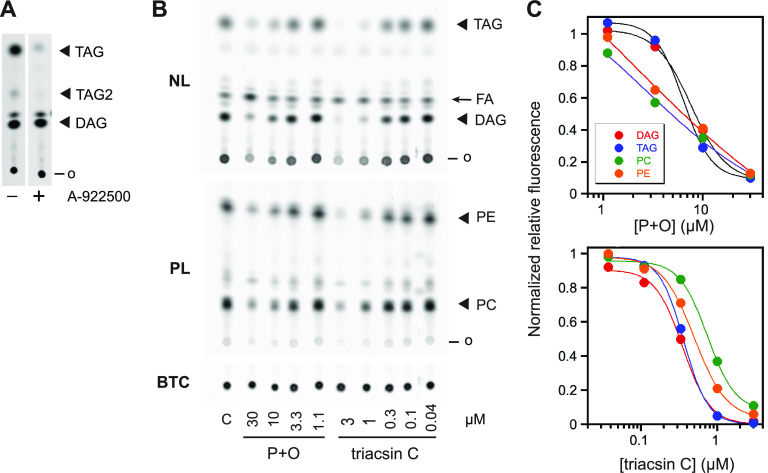
Assay validation. (A) Treatment with the diacylglycerol acyltransferase inhibitor A-922500 (10 μM) identifies TAG species that likely contain one (“TAG”) and two (“TAG2”) C4,C9-FA acyl chains. (B) Effects of equimolar mixtures of palmitate and oleate (“*P + O*,” concentration of each is indicated in μM) or triacsin C (μM) on C4,C9-FA incorporation into parasite neutral and polar lipids. FA, C4,C9-FA; o, origin. (C) Plots of BTC-normalized fluorescence intensities for the images in (B), expressed as a ratio of those for a no-additive control. Lines are nonlinear regression fits to a four-parameter sigmoidal curve. Data are representative of two independent experiments that yielded similar results.

Because the BODIPY fluorophore alters the mobility of C4,C9-FA-labeled lipids on silica TLC plates, unlabeled standards cannot be used to confirm lipid identities. We therefore used a potent inhibitor of mammalian diacylglycerol acyltransferase, A-922500 (31), to identify the major neutral lipid species with the highest mobility as triacylglycerol (TAG) (Fig. 3A). A minor species with lower mobility (TAG2 in Fig. 3A) is also depleted in the presence of A-922500; this likely represents the presence of an additional C4,C9-FA acyl group (i.e., double labeled *versus* single labeled). The remaining neutral lipid species is presumed to be diacylglycerol (DAG), as an abundance of evidence indicates that DAG and TAG are the two major neutral lipids synthesized by intraerythrocytic P. falciparum (4, 11, 32). The assignment of the two major polar lipid species as PC and PE is based on their abundance in parasite membranes (~45% and 35% of total phospholipids, respectively) and the relative mobilities of PC and PE. This assignment is consistent with prior studies with radiolabeled fatty acids demonstrating that PC and PE are the most rapidly synthesized phospholipids in asexual P. falciparum (4, 33).

To determine whether the lipid labeling observed with C4,C9-FA reflects physiological processes, we asked whether natural fatty acids compete with C4,C9-FA for lipid incorporation. Labeling reactions were carried out in the presence of various concentrations of an equimolar mixture of palmitate and oleate, as these two fatty acids are sufficient to sustain parasite growth (23). C4,C9-FA labeling was reduced in a concentration-dependent fashion and 30 μM each palmitate and oleate was sufficient to strongly suppress the incorporation of C4,C9-FA (also provided at 30 μM) into the major neutral and phospholipid species (Fig. 3B, C).

To confirm that C4,C9-FA is activated to the acyl-CoA thioester *in situ*, we examined the effects of the acyl-CoA synthetase inhibitor triacsin C (34) on lipid labeling. Triacsin C effectively inhibited labeling of neutral and polar lipids at low micromolar concentrations (Fig. 3B, C).

### Identification of cell-permeable inhibitors of glycerolipid biosynthesis.

We next sought to employ C4,C9-FA labeling to identify cell-permeable inhibitors of glycerolipid biosynthesis from two compound collections. First, we screened 17 potent, well-validated inhibitors of the mammalian lipid-metabolizing enzymes fatty acid amide hydrolase (FAAH) and monoacylglycerol lipase (MAGL) (Table S1). The rationale for this choice was based on the idea that compounds that bind tightly to FAAH and MAGL active sites may exhibit affinity for lipid- or fatty acid-metabolizing enzymes in P. falciparum, particularly those that act on monoacylated esters. Most are mechanism-based, covalent inhibitors.

**FIG 4:**
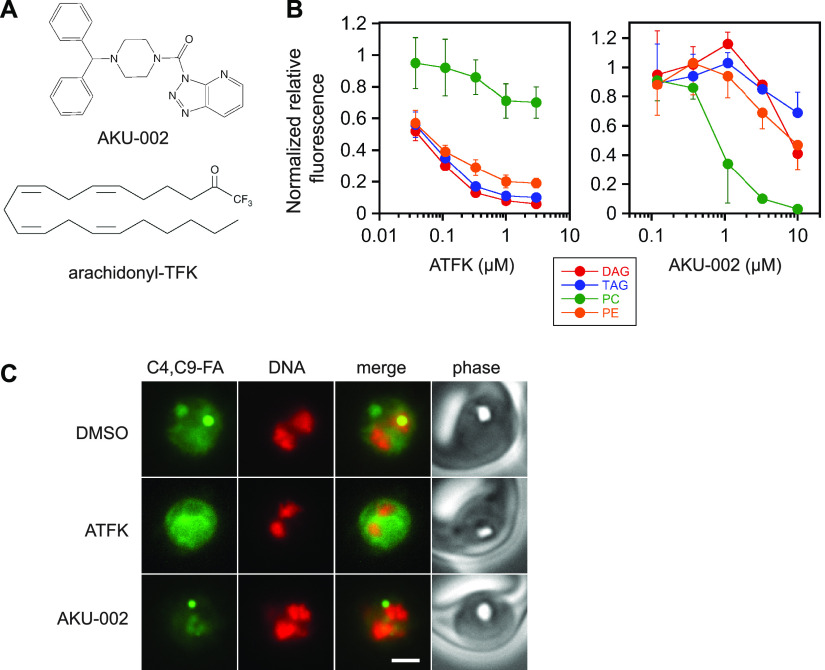
Effects of ATFK and AKU-002 on C4,C9-FA lipid labeling. (A) Compound structures. (B) Concentration dependence of inhibition of C4,C9-FA incorporation into DAG, TAG, PC and PE. BTC-normalized fluorescence values are expressed as a fraction of the no-inhibitor control. Data points are means from three independent experiments with standard deviation represented with bars. (C) Live-cell images of parasites labeled with C4,C9-FA in the presence of DMSO vehicle, 10 μM ATFK, or 10 μM AKU-002. DNA was stained with Hoechst 33342 and is pseudocolored red. Scale bar, 3 μm.

FAAH and MAGL inhibitors were screened at 10 μM concentration using the standard protocol outlined in Fig. 2. Three compounds reduced label incorporation by ≥95% in at least one lipid species: the MAGL inhibitor AKU-002 and the FAAH inhibitors PF-04457845 and arachidonyl trifluoromethylketone (ATFK) (see Table S1 for inhibition data). Surprisingly, each of these compounds appeared to exhibit some specificity for reduction of either neutral or polar lipids. AKU-002 preferentially suppressed PC synthesis, while PF-04457845 and ATFK suppressed neutral lipid synthesis much more effectively than that of phospholipids. To investigate these phenomena in more detail, we selected one compound of each type (AKU-002 and ATFK) for characterization of concentration dependence (Fig. 4A). ATFK inhibited the incorporation of C4,C9-FA into DAG, TAG and PE with a similar concentration dependence; in contrast, incorporation into PC was blocked much less efficiently (Fig. 4B). AKU-002 inverted this relationship, with C4,C9-FA incorporation into PC inhibited substantially more effectively in the 1–3 μM range. To assess the effects of the inhibitors on label distribution within the parasite, we imaged live parasites treated with 10 μM each compound (Fig. 4C). ATFK treatment greatly reduced the fluorescence intensity associated with the lipid droplet, while internal membrane fluorescence was largely preserved. In contrast, AKU-002 treatment strongly reduced the diffuse fluorescent signal within the parasite, which is consistent with a selective diminishment of PC.

**FIG 5:**
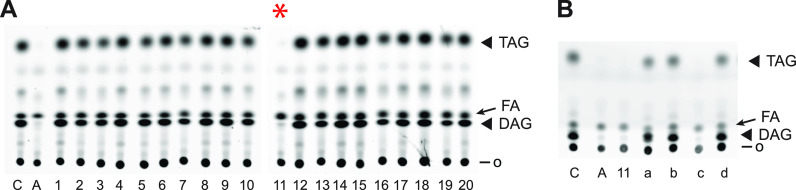
Identification of an inhibitor of P. falciparum lipid synthesis from the Malaria Box. (A) Screen of 20 pools representing 71 Malaria Box compounds for inhibition of parasite neutral lipid synthesis. C, no inhibitor control; A, ATFK (10 μM) positive control for inhibition. o, origin; FA, C4,C9-FA. The red asterisk indicates inhibition by pool 11. (B) Identification of the active compound in pool 11. a, b, c, d represent the four compounds in the pool; compound c is MMV665915.

The Malaria Box is a curated collection of 400 compounds with anti-malarial activity that was assembled to stimulate research into novel mechanisms of action (35). The collection is divided into five plates of 80 compounds each. We elected to screen plate A, which contains the most potent anti-malarial compounds. Due to depletion of some compounds used for prior screening studies, 71 plate A compounds were available. To efficiently screen the 71 compounds, we first created 20 pools of 3 to 4 compounds, each at 2.5 μM (the composition of the pools is provided in Table S2). Screening of these yielded one pool (number 11) that was effective in suppressing C4,C9-FA incorporation into neutral lipids (Fig. 5A). None of the pools exhibited a marked effect on phospholipid synthesis (not shown). To identify the active compound in pool 11, each of the four was assayed individually (Fig. 5B), resulting in identification of the compound MMV665915 (Fig. S5) as a potent inhibitor of C4,C9-FA incorporation into neutral lipids.

## DISCUSSION

We have shown that fluorescent fatty acid probes can be effective tools for interrogating lipid biosynthesis in intact, P. falciparum-infected erythrocytes. Of the various commercially available probes, C4,C9-FA was characterized in detail and was found to have several favorable attributes: rapid uptake and incorporation into parasite neutral and polar lipids, a physiological coenzyme A activation pathway, and good spectral and photostability properties that permit facile quantitation of fluorescent signal on TLC plates and live-cell microscopic imaging of membrane- and lipid droplet-associated lipids. While the steric bulk of the BODIPY group of C4,C9-FA likely alters the nature of its interactions with lipid metabolic enzymes (i.e., K_m_ and k_cat_), there is clearly sufficient flux to label the major lipid species. One caveat to the studies presented here is that these major species have not been unambiguously identified due to the unavailability of C4,C9-FA-labeled standards. We have used a diacylglycerol acyltransferase inhibitor to identify labeled TAG. The assignments of DAG, PC and PE are consistent with decades of research identifying these as highly abundant lipids in asexual, intraerythrocytic *Plasmodium* species. An outstanding question concerns the number of C4,C9-FA acyl chains per lipid molecule. We speculate that the most abundantly labeled phospholipids possess a single C4,C9-FA acyl group, as each additional group would further reduce the mobility of the lipid on the TLC plate and there is no evidence for a shift from double- to single-labeled PC or PE upon addition of competitor palmitate and oleate (Fig. 3B).

These studies were motivated by an interest in developing a live cell-based screen of inhibitors of lipid synthesis. We have demonstrated that our assay can identify inhibitors that are cell permeable, capable of blocking lipid metabolism *in situ*, and active at low micromolar concentrations from relatively small, targeted compound collections. In principle, the assay is highly versatile and should be responsive to inhibition of fatty acid uptake, formation of acyl-CoA, acyl group transfer during Kennedy pathway synthesis of DAG, PC and PE, and acylation of DAG to TAG. The utility of this approach was illustrated by identifying an inhibitor of lipid synthesis in the Malaria Box, MMV665915. It is unlikely that the observed inhibition is a nonspecific consequence of cytotoxicity; the inhibitor treatment period was limited to 1 h, and in principle all compounds of the Malaria Box are cytotoxic to the parasite, albeit with distinct mechanisms and kill rates, yet only one of 71 inhibited lipid synthesis. Interestingly, this compound shares structural similarities with a molecule (MMV019719) with anti-malarial activity that elicits resistance mutations in P. falciparum acyl-CoA synthetase (ACS) 11 (36) (Fig. S5). Thus, a plausible hypothesis is that MMV665915 exerts its effects through inhibition of ACS11, which has been annotated as an “essential” protein in a transposon mutagenesis study (37). Little else is known about this enzyme’s specific role in the parasite. Looking to the future, we anticipate that the discovery of inhibitors of fatty acid uptake can be combined with resistance selection and genome sequencing to identify key lipid metabolic enzymes that are amenable to dysregulation by cell-permeable small molecules.

Identification of arachidonyl trifluoromethylketone as an inhibitor of parasite lipid synthesis is in hindsight unsurprising; although this fatty acid analog is known as an FAAH inhibitor, it may also be a competitive inhibitor of parasite ACS given its structural similarity to endogenous fatty acids and its inability to react with ATP in the ACS active site. Its relative ineffectiveness in suppressing PC synthesis compared to PE, DAG, and TAG is an intriguing puzzle. One possible explanation is that low residual levels of C4,C9-FA-CoA are privileged for PC synthesis. Another is that there is a pathway of PC synthesis akin to the Lands cycle that relies on an ACS that is less effectively inhibited. Further study will be required to resolve this question.

Most surprising was the identification of the triazolopyridine urea inhibitor AKU-002 as a P. falciparum lipid synthesis inhibitor with a tendency to preferentially block PC synthesis. AKU-002 is a highly potent mammalian MAGL inhibitor (IC_50_ of ~ 1 nM) (38) but high potency is clearly not sufficient for the effect observed here, as the related triazole urea compound AKU-005 is more potent against MAGL (38) yet much less effective in suppressing incorporation of C4,C9-FA into PC. MAGL-like serine hydrolases have been identified in P. falciparum (39, 40); however, it is unclear how inhibiting an MAGL-like activity would suppress PC synthesis. Other possibilities include inhibition of a phospholipase involved in Lands cycle PC remodeling (although none has been identified to date) or inhibition of a lipid metabolic enzyme outside the serine hydrolase superfamily. While it is clear that AKU-002 has a distinct mechanism of action, elucidation of that mechanism will require further study.

## MATERIALS AND METHODS

### Chemicals.

BODIPY 500/510 C_4_,C_9_ (abbreviated as “C4,C9-FA”), BODIPY 500/510 C_1_,C_12_, BODIPY FL C_16_, BODIPY FL C_5_, BODIPY TR ceramide (abbreviated as “BTC”), and HCS LipidTox Red neutral lipid stain were obtained from Thermo Fisher. C11-TOPFLUOR was from Avanti Polar Lipids. A-922500 and fatty acid amide hydrolase inhibitors were obtained from Cayman Chemical. Piperazine-based monoacylglycerol lipase inhibitors (38) were a gift of Tapio Nevalainen, University of Eastern Finland. The Malaria Box (35) was a gift from the Medicines for Malaria Venture. Fatty acid-free bovine serum albumin was obtained from SigmaAldrich (product number A7511).

### Parasite culture.

P. falciparum clone 3D7 was used for all experiments and was cultured in O^+^ human erythrocytes (Interstate Blood Bank) at 2% hematocrit in RPMI 1640 supplemented with 27 mM sodium bicarbonate, 11 mM glucose, 0.37 mM hypoxanthine, 10 μg/mL gentamicin and 5 g/L Albumax I (Invitrogen). Cultures were incubated at 37°C in a 5% CO_2_ environment and were synchronized by treatment with 5% sorbitol. For fatty acid uptake experiments, multiple sorbitol treatments were employed to generate a culture in which trophozoite-stage parasites predominated (defined as 28 to 36 h post-invasion, with an ~42 h replication cycle time for the 3D7 line). Parasites in this window of synchrony were characterized by a large hemozoin crystal, an elliptical shape, and 1 to 4 nuclei.

### Standard dual labeling protocol.

A parasite culture containing trophozoites synchronized as described above at ~10% parasitemia was pelleted by centrifugation, resuspended in prewarmed complete RPMI containing 1 μM BTC at 4% hematocrit, and incubated for 1 h. The culture was washed twice in incomplete RPMI (i.e., lacking Albumax I), resuspended in incomplete RPMI at 8% hematocrit, and aliquots of 0.5 mL were transferred to a 24-well plate. In adjacent wells were placed 0.5 mL of incomplete RPMI supplemented with 60 μM C4,C9-FA and 4 mg/mL fatty acid-free BSA (i.e., at 2× concentration). For competition assays, equimolar mixtures of palmitic and oleic acids were also added to the C4,C9-FA/BSA solution at 2× concentration. For inhibition assays, inhibitors were added at 1× concentration from 1000× DMSO stocks to both the parasite culture and the probe-containing media. Cultures were incubated for 30 min. To initiate labeling, probe-containing media were added to cultures, followed by a 30 min incubation. All incubation steps were conducted at 37°C in a CO_2_ incubator with gentle mixing on an orbital rotator. Labeling was quenched by transferring each 1 mL culture to 9 mL of ice-cold 0.03% saponin in Dulbecco’s phosphate-buffered saline (PBS). After 5 min on ice, parasites were harvested by centrifugation at 1940 × *g* at 4°C for 10 min and were washed twice with cold PBS. Parasite pellets were stored at −80°C.

### Fluorescence imaging and lipid droplet labeling.

Live parasites were imaged by transferring 25 μL of labeled parasite culture to 1 mL of cold complete RPMI to quench labeling. Wet mounts of cultures were imaged using a Zeiss Axioimager M1 microscope with a 100×/1.4 NA objective and an Axiocam MRm camera. To label lipid droplets, the BODIPY-TR-ceramide step was omitted and parasites were labeled with C4,C9-FA as described above. Cultures were fixed with 4% formaldehyde/0.01% glutaraldehyde for 30 min at room temperature, washed with PBS, and incubated with a 250-fold dilution of HCS LipidTox Red in PBS for 1 h at room temperature. Contrast of all images was adjusted with Adobe Photoshop. Pearson’s correlation coefficient was determined using the “Coloc 2” tool in Fiji (ImageJ) after placing a circular region of interest around the putative lipid droplet.

### Lipid isolation and analysis by thin-layer chromatography (TLC).

Saponin-isolated parasite pellets were resuspended in 188 μL of chloroform/methanol (1:2 ratio) and vortexed vigorously for 15 min. 62.5 μL chloroform and then 62.5 μL water were added with brief mixing. Samples were centrifuged at 1000 × *g* for 1 min and the organic layer was transferred to a new tube. 100 μL chloroform was added to the aqueous layer and vortexed. The organic layers were combined, and solvent was evaporated in a vacuum centrifuge. Samples were dissolved in 20 to 30 μL chloroform and 1 μL was spotted on a glass HPTLC Silica Gel 60 plate (EMD Millipore, product number 1.05633.0001). Neutral lipids were developed with 40:60:1 heptane/diethyl ether/acetic acid. Plates were imaged on a Typhoon Trio imager using a 532 nm laser and a 580/30 bandpass filter for green C4,C9-FA fluorescence and a 633 nm laser and a 670/30 bandpass filter for red BTC fluorescence. Polar lipids were then developed on the same plate with 10:4:1 chloroform/methanol/water and imaged as above. We note that this tandem development procedure frequently led to the appearance of neutral lipid “shadows” due to incomplete elution of neutral lipid species during the polar lipid development (e.g., see Fig. 1D); however, these generally did not interfere with polar lipid quantitation. BTC and neutral and polar lipid fluorescence intensities were quantified using ImageQuantTL software (GE Biosciences). BTC fluorescence intensities from the neutral lipid separation were used to normalize both neutral and polar lipid fluorescence intensities.

### Malaria Box inhibition assays.

Twenty pools of three or four Malaria Box “Plate A” compounds (comprising a total of 71 compounds) were generated with each compound at 2.5 mM and assayed at 2.5 μM; see Table S2 for the composition of each pool. Pools were tested for inhibition of lipid synthesis according to the standard protocol.
