# Supplementary material for: Leveraging a Fluorescent Fatty Acid Probe to Discover Cell-Permeable Inhibitors of Plasmodium falciparum Glycerolipid Biosynthesis
Source: Microbiol Spectr. 2022 Oct 31;10(6):e02456-22. doi: 10.1128/spectrum.02456-22 (PMC9769509; doi:10.1128/spectrum.02456-22)
Supplement: Supplemental file 1 — Supplemental material. Download spectrum.02456-22-s0001.pdf, PDF file, 2.2 MB [file spectrum.02456-22-s0001.pdf]

Supplemental Figure S1: Structures of fluorescent probes used in this study.

BODIPY-C4,C9
Tradename: BODIPY™ 500/510 C_4, C_9
Chemical name: 5-butyl-4,4-difluoro-4-bora-3a,4a-diaza-*s*-Indacene-3-nonanoic acid
Source: ThermoFisher B3824

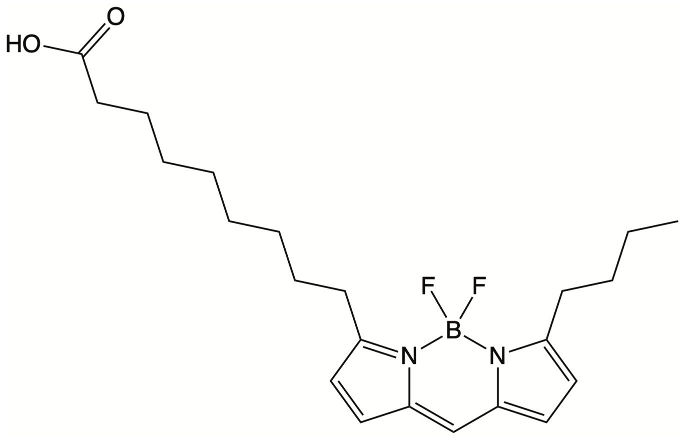

BODIPY-C1,C12
Tradename: BODIPY™ 500/510 C_1, C_{12}
Chemical name: 4,4-difluoro-5-methyl-4-bora-3a,4a-diaza-*s*-indacene-3-dodecanoic acid
Source: ThermoFisher D3823

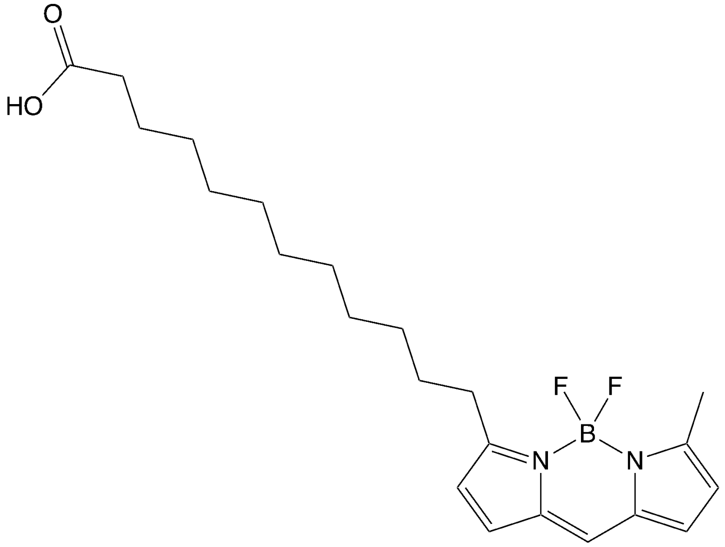

BODIPY-C5
Tradename: BODIPY™ FL C_5
Chemical name: 4,4-difluoro-5,7-dimethyl-4-bora-3a,4a-diaza-*s*-indacene-3-pentanoic acid
Source: ThermoFisher D3834

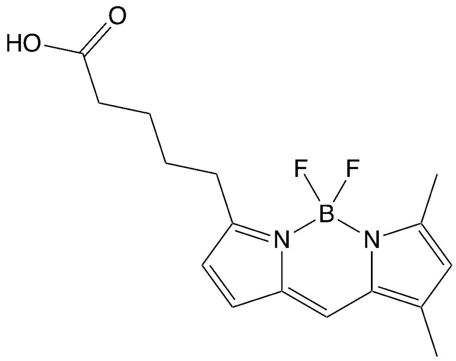

BODIPY-C16
Tradename: BODIPY™ FL C_{16}
Chemical name: 4,4-difluoro-5,7-dimethyl-4-bora-3a,4a-diaza-*s*-indacene-3-hexadecanoic acid
Source: ThermoFisher D3821

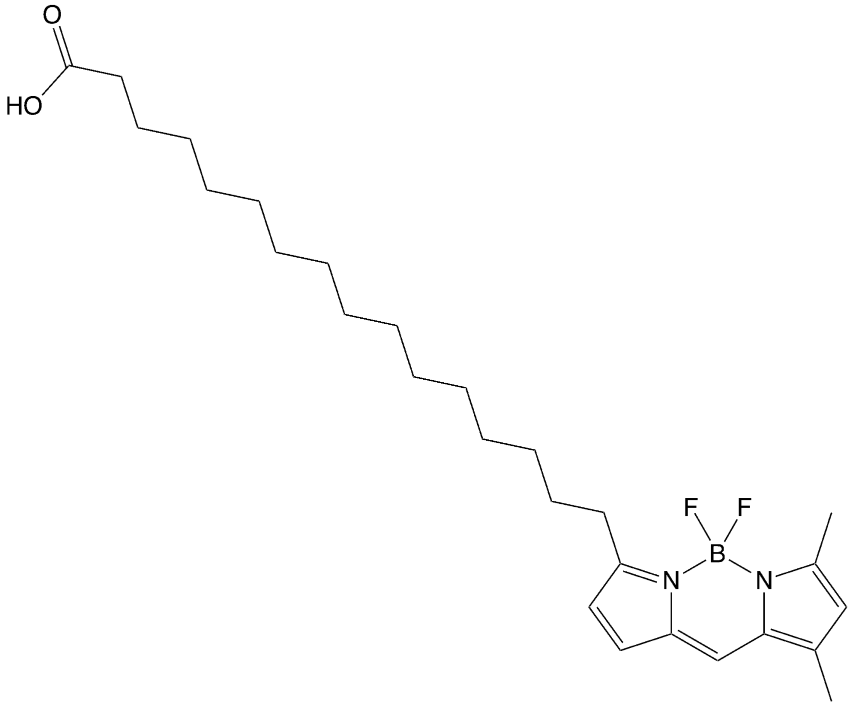

TOPFLUOR-C11
Chemical name: 11-(dipyrrometheneboron difluoride)undecanoic acid
Source: Avanti Polar Lipids

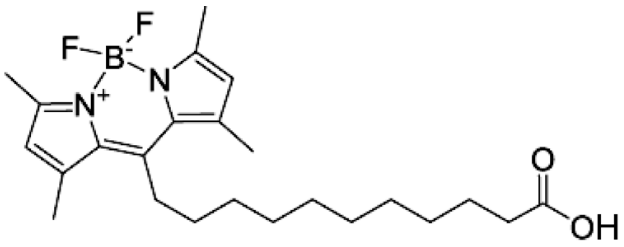

BODIPY-TR-CERAMIDE
Source: ThermoFisher D7540

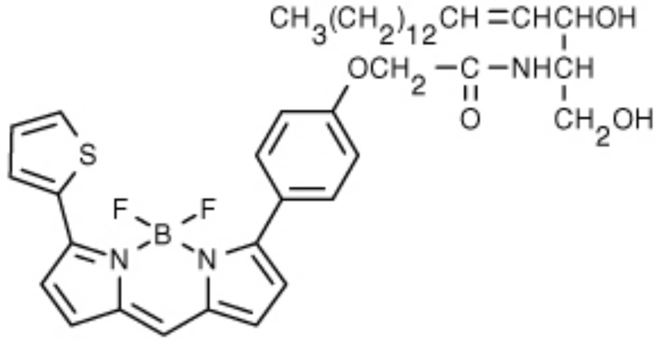

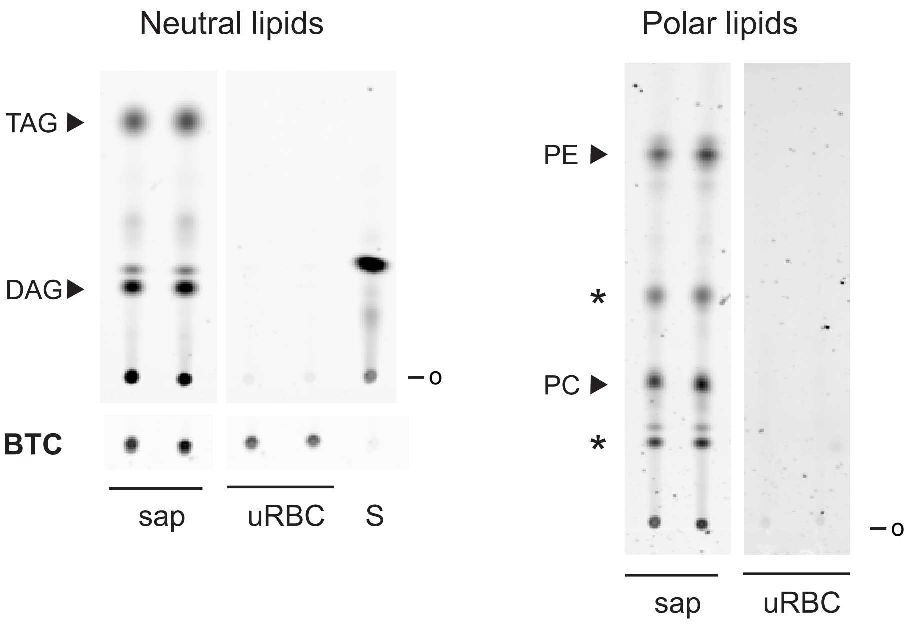

Supplemental Figure S2: Comparison of C4,C9-FA uptake by equal numbers of saponin-isolated parasites (”sap”) and uninfected erythrocytes (”uRBC”), each labeled and extracted in duplicate. S, C4,C9-FA standard. Asterisks indicate residual neutral lipids (DAG and TAG) in the polar lipid image. o, origin.

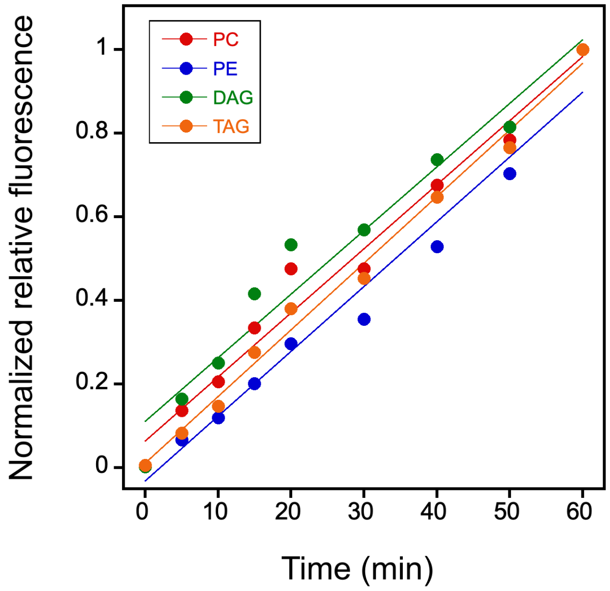

Supplemental Figure S3: BTC-normalized fluorescence intensities over time for the major lipid species in Fig. 1B, expressed as a fraction relative to the values at 60 minutes. Lines are linear regression fits.

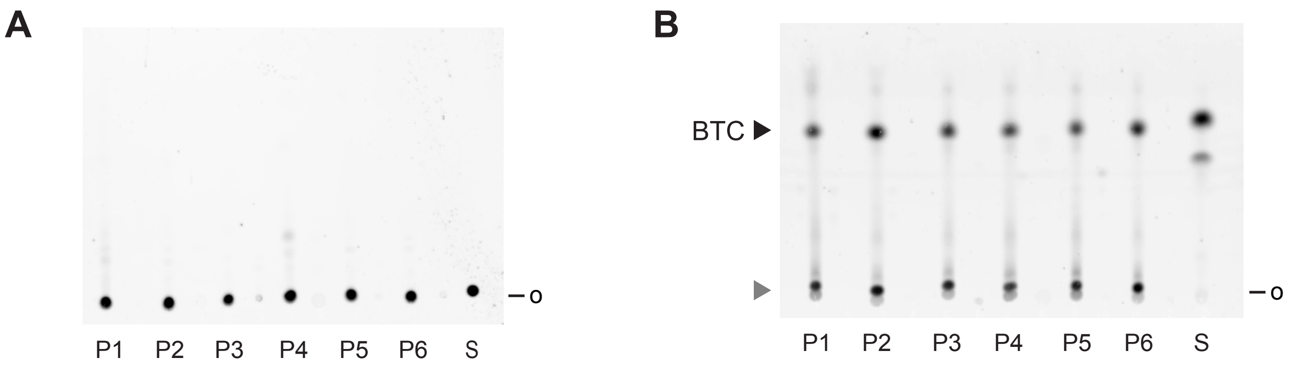

Supplemental Figure S4: Migration of BTC after TLC development of (A) neutral lipids and (B) polar lipids. P1-P6 are parasite lipid extracts and S is a BTC standard. o, origin. In (B), BTC is indicated with a black arrowhead and a polar metabolite (probably sphingomyelin) with a gray arrowhead. The TLC images correspond to those shown in Fig. 1A.

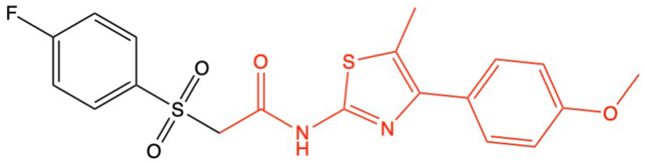

MMV665915

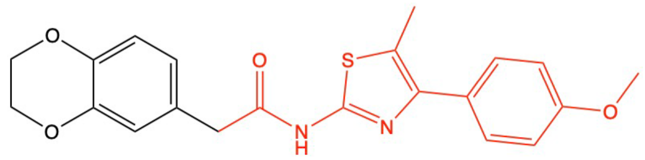

MMV019719

Supplemental Figure S5: Structures of the Malaria Box compound MMV665915, identified as an inhibitor of neutral lIipid synthesis in this study, and the compound MMV019719 that was found to induce mutations in ACS11 in the resistance selection study of Cowell *et al* (2018). Structural features common to both are shown in red.

Supplemental Table S1: Inhibition of C4,C9-FA labeling of neutral- and phospho-lipids by FAAH and MAGL inhibitors (10 μM concentration). Values are BTC-normalized and are reported as a percentage of no-inhibitor control. Where three independent assays were conducted, values are reported as means ± standard deviation. For compounds with fewer than three replicates, individual values are provided. Compounds indicated in bold font exhibit ≥95% inhibition of at least one lipid species. SH, serine hydrolase; MAGL, monoacylglycerol lipase; FAAH, fatty acid amide hydrolase. CID, compound ID.

Inhibitor	Pubchem CID	Source	Mechanism	DAG	TAG	PC	PE
MAGL inhibitors							
**AKU-002**	**none**	**T. Nevalainen**	**covalent SH**	**51 ± 15**	**83 ± 19**	**1.6 ± 1.1**	**28 ± 7**
AKU-005	none	T. Nevalainen	covalent SH	106, 75	136, 80	40, 37	76, 58
AKU-006	none	T. Nevalainen	covalent SH	59, 40	62, 43	25, 36	46, 35
AKU-010	none	T. Nevalainen	covalent SH	90, 73	109, 82	65, 70	83, 71
JJKK-006	none	T. Nevalainen	covalent SH	60	74	10	38
JJKK-033	none	T. Nevalainen	covalent SH	88, 61	91, 61	43,38	63, 48
JJKK-046	none	T. Nevalainen	covalent SH	64	76	87	63
JJKK-048	none	T. Nevalainen	covalent SH	86, 61	95, 74	72, 53	65, 55
FAAH inhibitors							
PF-3845	25154867	Cayman	covalent SH	6, 5	7, 7	57, 51	32, 25
**PF-04457845**	**24771824**	**Cayman**	**covalent SH**	**3 ± 1**	**5 ± 4**	**50 ± 14**	**23 ± 9**
PF-622	11515763	Cayman	covalent SH	104, 91	113, 87	106, 101	113, 109
PF-750	25154868	Cayman	covalent SH	80	89	97	95
JP83	35027739	Cayman	covalent SH	17, 14	18, 18	53, 63	31, 32
JP104	24860363	Cayman	covalent SH	8	9	52	26
SA57	44589122	Cayman	covalent SH	67, 55	75, 60	89, 102	80, 81
URB597	1383884	Cayman	covalent SH	16, 4	21, 6	82, 35	51, 22
**arachidonyl-TFK**	**5280436**	**Cayman**	**non-covalent**	**1 ± 1**	**1 ± 1**	**59 ± 9**	**24 ± 5**

Supplemental Table S2: Compound pools created to screen Plate A of the Malaria Box collection.

Pool	Compounds (MMV-)
1	019066<br>006427<br>006203<br>666101
2	011099<br>007116<br>007839
3	665941<br>666062<br>665977<br>666596
4	000642<br>009063<br>007384<br>000662
5	020885<br>020549<br>396679
6	666600<br>006558<br>020548<br>396678
7	000570<br>001246<br>396797
8	006172<br>007160<br>019258<br>006861
9	008294<br>020439<br>008138
10	006309<br>006429<br>007686<br>006457

Pool	Compounds (MMV-)
11	011259<br>396672<br>665915<br>665916
12	006087<br>396703<br>011256<br>396693
13	019406<br>019871<br>020788
14	020492<br>006937<br>666693
15	006278<br>085583<br>000448<br>666691
16	006455<br>008956<br>007907
17	008416<br>020500<br>665785
18	665782<br>665820<br>665827<br>665805
19	019110<br>665874<br>665878<br>665831
20	665876<br>001038<br>666021
